# Assessment of a Therapeutic X-ray Radiation Dose Measurement System Based on a Flexible Copper Indium Gallium Selenide Solar Cell

**DOI:** 10.3390/s22155819

**Published:** 2022-08-04

**Authors:** Dong-Seok Shin, Tae-Ho Kim, Jeong-Eun Rah, Dohyeon Kim, Hye Jeong Yang, Se Byeong Lee, Young Kyung Lim, Jonghwi Jeong, Haksoo Kim, Dongho Shin, Jaeman Son

**Affiliations:** 1 Proton Therapy Center, National Cancer Center, Goyang 10408, Korea; 2 Department of Radiation Oncology, Myongji Hospital, Goyang 10475, Korea; 3 Department of Radiation Oncology, Seoul National University Hospital, Seoul 03080, Korea

**Keywords:** flexible dosimeter, copper indium gallium selenide solar cell, radiation therapy

## Abstract

Several detectors have been developed to measure radiation doses during radiotherapy. However, most detectors are not flexible. Consequently, the airgaps between the patient surface and detector could reduce the measurement accuracy. Thus, this study proposes a dose measurement system based on a flexible copper indium gallium selenide (CIGS) solar cell. Our system comprises a customized CIGS solar cell (with a size 10 × 10 cm^2^ and thickness 0.33 mm), voltage amplifier, data acquisition module, and laptop with in-house software. In the study, the dosimetric characteristics, such as dose linearity, dose rate independence, energy independence, and field size output, of the dose measurement system in therapeutic X-ray radiation were quantified. For dose linearity, the slope of the linear fitted curve and the R-square value were 1.00 and 0.9999, respectively. The differences in the measured signals according to changes in the dose rates and photon energies were <2% and <3%, respectively. The field size output measured using our system exhibited a substantial increase as the field size increased, contrary to that measured using the ion chamber/film. Our findings demonstrate that our system has good dosimetric characteristics as a flexible in vivo dosimeter. Furthermore, the size and shape of the solar cell can be easily customized, which is an advantage over other flexible dosimeters based on an a-Si solar cell.

## 1. Introduction

Radiation therapy is one of the primary treatments for cancer. Recently, various radiation therapy techniques such as image-guided radiotherapy and adaptive radiotherapy have been adopted by clinics to deliver highly conformal doses to tumors while sparing normal tissues [1,2,3,4].

Despite the accuracy of these techniques, accidents (higher dosage to normal tissues) and incidents (under dosage to tumors) can occur [5,6]. These accidents/incidents could be caused by various reasons such as patient motion/dose calculation errors and lead to radiation-induced side effects in normal tissues and low effectiveness of radiation therapy [5,6,7,8,9]. Therefore, the radiation doses delivered to the patient during treatment should be measured and monitored. The doses can be measured by attaching detectors to skins (non-invasive method) or as close as possible to the tumor/organ at risk (invasive method). The non-invasive method obtains doses delivered to the inside of the patient using a relation between the entrance and exit doses (which are measured at skins) [10]. In general, in external beam radiotherapy, the non-invasive method is preferred. The process of dose measurements (including both non-invasive and invasive methods) during treatment is typically called “in vivo dosimetry” in the field of radiation therapy [11,12,13,14].

Many clinics have used various types of detectors for patient-specific quality assurance (QA), daily/monthly/annual machine QA, beam monitoring, and in vivo dosimetry. For patient-specific QA (which is to compare the planned dose distribution with the measured dose distribution before patient treatment), commercialized two-dimensional (2D) detectors (such as radiochromic/gafchromic films, ion chamber array, and diode array) have been used [15,16,17,18]. Although the films possess advantages such as high spatial resolution and tissue equivalency, film-scanner performance can affect the accuracy of film dosimetry. Contrastively, the 2D array detectors do not require additional processes such as film scanning. However, they have low resolutions (such as 7.62 and 5.00 mm) and are not tissue equivalent. To improve the limitations, several studies have proposed 2D scintillators and 2D optical fiber detectors [19,20]. Recently, a three-dimensional (3D) detector (such as a polymer gel dosimeter) has been suggested to improve the accuracy of patient-specific QA [21,22]. Additionally, an electronic portal imaging device (EPID), which is mounted to a linear accelerator machine, and a log file (related to dynamic multi-leaf collimator) based method have been used to improve the time efficiency of patient-specific QA [23,24]. For daily/monthly/annual machine QAs (which are procedures to ensure that the machine characteristics do not deviate substantially from the baseline obtained at the time of acceptance test), generally, the cylindrical ion chamber is mainly used. This is because absorbed doses for megavoltage photon beams can be easily/accurately measured using the calibrated ion chamber according to TG-51 [25] or TRS-398 [26] reports. For beam monitoring, various transmission detectors such as plastic scintillators, diode arrays, and radioluminescence films have been developed and used [27,28,29]. For in vivo dosimetry, one-dimensional (1D) detectors such as thermoluminescent dosimeters (TLDs), optically stimulated luminescent dosimeters (OSLDs), and metal-oxide-semiconductor field-effect transistors (MOSFETs), as well as 2D film, have been used [30,31,32,33,34,35,36,37,38,39,40,41,42]. This is due to the bulky dimensions of 2D/3D detectors (except for film) that make it difficult to stably place them on the skins or nature orifices of the patient. To solve this limitation, recently, 2D/3D in vivo dosimetry methods based on EPID have been proposed [43,44]. However, the EPID dosimetry requires dose reconstruction algorithms such as back-projection since EPID measures transit dose (not “absorbed dose” of the patient). This means the accuracy of in vivo EPID dosimetry depends on the performance of the dose reconstruction algorithm. Moreover, EPID dosimetry cannot be applied to special radiotherapy techniques with large field sizes/long source-to-surface distance, such as total body irradiation (TBI), which is the process of uniformly irradiating the patient’s entire body before stem cell transplantation [45,46,47]. Therefore, 1D detectors or 2D film have been typically used in many clinics for in vivo dosimetry of the TBI.

Among the 1D detectors, TLDs have good characteristics for in vivo dosimetry, such as small size, energy independence, and tissue equivalency [30,31]. However, pre- and post-processing are required to convert thermoluminescent signals to doses, which is extremely time-consuming. Such detectors are called passive detectors. OSLDs are similar to TLDs, except for a few differences such as the use of light instead of heat to read out luminescent signals [32,33,34,35]. Finally, MOSFETs are real-time dosimeters, i.e., active dosimeters with high sensitivity and small volumes [36,37,38,39,40]. However, they have limited lifetimes. For 2D film detectors, high resolution and tissue equivalency are good characteristics [41,42]. However, scan processing is required (passive detector), which increases the workload. In addition, the accuracy of dosimetry can decrease by human error and depends on the performance of the scanner.

Although these in vivo dosimeters for the TBI (TLDs, OSLDs, MOSFETs, film) have good properties, their lack of flexibility is a major disadvantage. Consequently, gaps cannot be avoided when attaching the dosimeter to a curved surface on a patient, which can cause inaccurate dose measurements. Therefore, flexible dosimeters for the TBI in vivo dosimetry are required to obtain accurate measurements.

A promising candidate for flexible dosimeters is the thin-film solar cell, such as amorphous silicon (a-Si), cadmium telluride (CdTe), copper indium gallium selenide (CIGS), and perovskite solar cells [48,49,50,51,52]. Several studies have proposed flexible dosimeters based on a-Si [49,50] and perovskite solar cells [52] for photon beams. Zygmanske et al. developed a flexible dosimeter for megavoltage (MV)/kilovoltage photon beams using eight a-Si solar cells [49]. The authors demonstrated that it had good properties such as dose linearity, dose rate independence, and minor energy dependence. However, the a-Si solar cells typically have extremely low efficiency (ratio of output energy from input energy), i.e., up to 10.2% [53], compared with other solar cells. Consequently, measurements obtained under low dose rates, i.e., 5–100 MU/min, (low input energy to the solar cell) might be inaccurate. This is because the output of the solar cell could be lower than the resolution of the detecting device such as an electrometer. Recently, Jeong et al. improved the efficiency of a-Si solar cells by attaching a 1 mm thick scintillator screen to them [50]. However, this modification increases the thickness of the dosimeter and could reduce flexibility. Gill et al. proposed a flexible perovskite solar-cell detector for real-time X-ray measurements [52]. This detector exhibited a sensitivity approximately 5.5 times higher than that of a-Si solar cells. However, perovskite solar cells have not yet been commercialized and are not readily available. Consequently, adopting this method in clinical applications is difficult. Moreover, perovskite solar cells are extremely vulnerable to humidity, which could reduce the accuracy of dose measurements under high-humidity conditions, i.e., >50% relative humidity [54].

Recent advances in CIGS solar cell technology have enabled the manufacture of flexible CIGS solar cells, which are compound thin-film solar cells with high efficiency and stability comparable with silicon solar cells [53]. Additionally, they have a high radiation hardness [55,56,57] and have been commercialized [58]. Owing to these advantages, CIGS cells are suitable as flexible in vivo dosimeters for radiation therapy. However, only a limited number of related studies have been conducted to date [59,60].

Therefore, we propose a measurement system for therapeutic X-ray radiation based on flexible CIGS solar cells. This study demonstrates the configuration of the measurement system and quantifies its basic dosimetric characteristics such as dose linearity, dose rate independence, energy independence, and field size output.

## 2. Materials and Methods

### 2.1. System Configuration

**Figure 1:**
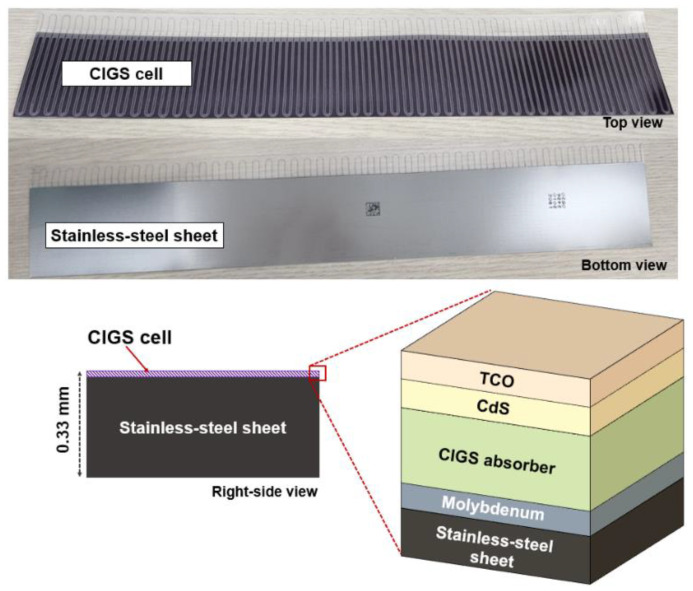
A commercialized copper indium gallium selenide (CIGS) solar cell and its structure. The area comprising the CIGS cell is the active area that converts therapeutic X-ray radiation to electrical signals. TCO and CdS denote transparent conductive oxide and cadmium sulfide, respectively.

Our system comprises a 0.33 mm thick CIGS solar cell (MiaSolé Hi-Tech Corp, Santa Clara, CA, USA), a voltage amplifier (IsoBlock V-4C, Verivolt, San Francisco, CA, USA), a data acquisition (DAQ) module (NI-9239/cDAQ-9189, NI, Austin, TX, USA), and a laptop with in-house software (Figure 1).

**Figure 2:**
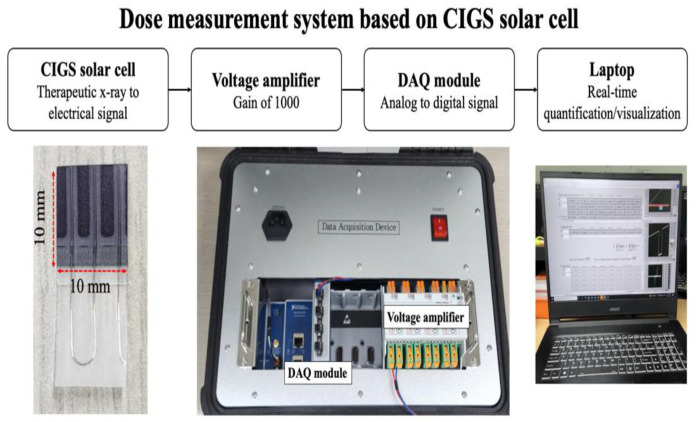
Configuration and data acquisition process of our measurement system based on a copper indium gallium selenide (CIGS) solar cell. In this system, therapeutic X-ray radiation is converted to electrical signals by the CIGS solar cell, and the electrical signals were digitalized through data acquisition (DAQ) module. The digitalized signals were visualized in real-time using a laptop with in-house software.

The CIGS solar cell selected for use in this study was composed of a stainless-steel sheet, molybdenum, absorber, cadmium sulfide, and transparent conductive oxide (Figure 2) and could be customized to obtain user-intended sizes and shapes [58]. Therefore, the CIGS solar cell was reduced from its original size (308 × 44 mm^2^) to a custom size (10 × 10 mm^2^), which is an empirically selected size (to evaluate their dosimetric characteristics as an in vivo dosimeter. The cut CIGS solar cell can convert therapeutic X-ray radiation into electrical signals. A voltage amplifier was used to improve the accuracy of signal acquisition under low intensity conditions, such as low dose rates. The electrical signals from the CIGS solar cells were amplified with a gain of 1000, and the DAQ module digitalized the amplified electrical signals with a sampling rate of ≤50 kHz. Finally, the digitalized signals were quantified and visualized using a laptop with in-house software. That is, in our system, continuous dose measurement is possible unless the CIGS solar cell is severely damaged by the radiation, or the laptop memory is insufficient. In this study, we defined “total signal intensity” as the sum of acquired signals by our system during X-ray radiation.

### 2.2. Quantifications and Evalutions of Dosimetric Characteristics

#### 2.2.1. Experimental Setup

**Figure 3:**
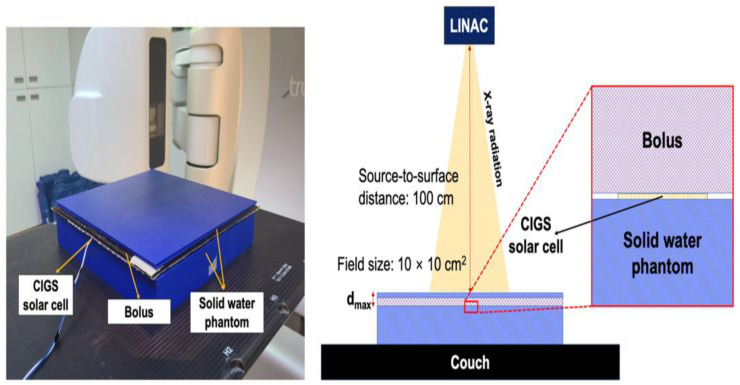
Experimental setup for quantifications/evaluations for dosimetric characteristics of measurement system based on a copper indium gallium selenide (CIGS) solar cell. A bolus (flexible/water equivalent material) was used instead of a solid water phantom to reduce airgaps between the solid water phantom and bolus. LINAC and d_max_ denote linear accelerator and depth at maximum dose, respectively.

The following dosimetric characteristics were quantified and evaluated: (a) dose linearity, (b) dose rate independence, (c) energy independence, and (d) field size output. For these quantifications, the CIGS solar cell was aligned at the beam isocenter and sandwiched between a bolus (flexible/water-equivalent material) and solid water phantom, as shown in Figure 3. The use of a bolus instead of a solid water phantom could increase the accuracy of dose measurement by minimizing the airgaps between the bolus and solid water phantom due to their flexibility. The CIGS solar cell was placed at a depth of d_max_, which is the depth at maximum dose, for all evaluations except for the field size output, which was measured at a depth of 10 cm. To reduce the signal noise caused by visible light, a black sheet was attached to the CIGS solar cell. Additionally, the signals from the dark currents were subtracted before every measurement. The source-to-surface distance (SSD) and sampling rate were set to 100 cm and 2 kHz, respectively, for all evaluations. The remaining experimental variables of dose, dose rate, energy, and field size were varied for each evaluation. Table 1 summarizes the values of the experimental variables for each evaluation. All quantifications and evaluations were repeated thrice.

#### 2.2.2. Dose Linearity

To evaluate dose linearity, different radiation doses were delivered to the CIGS solar cell using a TrueBeam linear accelerator (Varian Medical Systems, Palo Alto, CA, USA) as shown in Table 1. A field size of 10 × 10 cm^2^ and SSD of 100 cm were used. The acquired signals were normalized based on those with a dose of 1200 cGy, and then scaled 1200 times. The relationship between the mean total signal intensities (from our system) and radiation doses was visualized using the linear fitting method, and the coefficient of determination (R-square) was also calculated.

#### 2.2.3. Dose Rate Independence

To evaluate the dose rate independence, doses of 100 cGy were delivered to the CIGS solar cell at different dose rates from 100 to 600 MU/min (with an interval of 100 MU/min) using a TrueBeam linear accelerator. Most linear accelerators provide this range of dose rates, and most radiotherapies are performed in those. Six MV photon beams with a field size of 10 × 10 cm^2^ were used, and the SSD was set to 100 cm. The obtained signals were normalized to those at a dose rate of 600 MU/min (baseline). The baseline signals were compared with those of the other dose rates.

#### 2.2.4. Energy Independence

To evaluate energy independence, different photon beams were used (Table 1). The CIGS solar cell was placed at d_max_ depth (a depth at maximum dose) corresponding to each photon energy. Subsequently, the SSD and field size were set to 100 cm and 10 × 10 cm^2^, respectively. The CIGS solar cell was then irradiated with doses of 100 cGy at a dose rate of 600 MU/min. The obtained signals were normalized based on those for a 6 MV photon beam and compared.

#### 2.2.5. Field Size Output

To evaluate the signals (from the CIGS solar cell) according to field size, various field sizes (3 × 3, 4 × 4, 6 × 6, 8 × 8, 10 × 10, 12 × 12, 15 × 15, and 20 × 20 cm^2^) for a constant SSD of 100 cm were used. The CIGS solar cell was placed at a depth of 10 cm to minimize errors due to electron contamination (from the linear accelerator head) and irradiated with doses of 100 cGy using 6 MV photon beams. The acquired signals were normalized based on those obtained for a 10 × 10 cm^2^ field size. In addition, to compare the signals (CIGS) with baseline data, the field size output was measured using an ion chamber (Farmer Ionization Chamber 30013, PTW-Freiburg, Freiburg, Germany). The ion chamber was inserted into the solid water phantom hole (located in the center of the phantom) and aligned at the isocenter. The ion chamber was then placed at a depth of 10 cm based on the effective point of measurement (a shift of 0.6 × chamber cavity radius in upstream direction based on center axis) that corrects ionization gradient by cylindrical chamber geometry and irradiated under the same conditions. In addition, the same experiment was performed using EBT3 radiochromic film (Ashland Specialty Ingredients, Wayne, NJ, USA). The region of interest for the film was 0.7 × 0.7 cm^2^. An integrated signal was obtained by summing all intensities corresponding to this region of interest. Normalizations for chamber and film were performed in the same manner. The field size outputs (by ion chamber and film) were compared to those of the CIGS solar cell.

#### 2.2.6. Flexibility Test

To evaluate the flexibility, the signal of the CIGS solar cell in the bent condition was compared with that in the flat condition. The CIGS solar cell was bent at a curvature radius of 20 mm (0.05 mm^−1^ of curvature), which is comparable to the human surface curvature of the forehead, back of the hand, and thorax. The bent CIGS solar cell was sandwiched between 3D-printed custom slabs (including a speed bump-like shape) based on high-impact polystyrene material. The bent CIGS solar cell was placed at 1.3 cm depth in the custom slab phantoms. Subsequently, the CIGS solar cell was irradiated using a 6 MV photon beam with doses of 100 cGy at a dose rate of 600 MU/min and SSD of 100 cm. To compare the performance of the bent CIGS solar cell with that of the flat CIGS solar cell, the signal of the flat CIGS solar cell was obtained under the same experimental conditions. The obtained signals were normalized based on those of the flat CIGS solar cell.

## 3. Results

### 3.1. Dose Linearity

**Figure 4:**
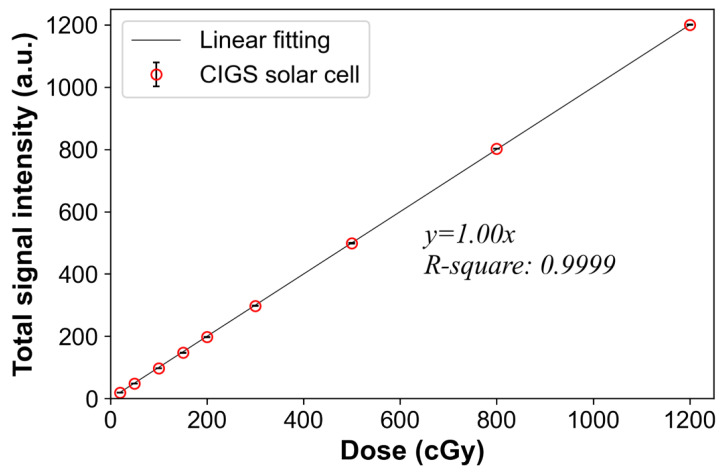
Relationship between the signals of the copper indium gallium selenide (CIGS) solar cell and doses delivered using 6 MV photon beams. Total signal intensity is the sum of the obtained signals from the CIGS solar cell during irradiation. The normalizations were performed based on the signal corresponding to a dose of 1200 cGy. Error bars denote the standard deviations between three repeated measurements.

Figure 4 shows the linear relationship between the signals of the CIGS solar cell and the doses. The standard deviations (error bars in Figure 4 and Table 2) between three repeated measurements approach zero, and the slope of the linear fitted curve and the coefficient of determination are 1.00 and 0.9999, respectively. These results demonstrate that our system, based on CIGS solar cells, has good dose linearity.

### 3.2. Dose Rate Independence

**Figure 5:**
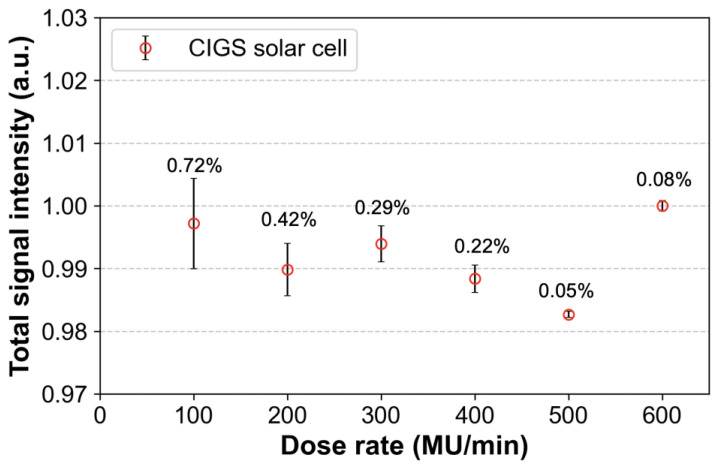
The signals obtained from the copper indium gallium selenide (CIGS) solar cell according to changes in dose rate from 100 to 600 MU/min at intervals of 100 MU/min. Total signal intensity is the sum of the obtained signals from the CIGS solar cell during irradiation. All the signals were normalized based on the signal corresponding to a dose rate of 600 MU/min. Error bars denote the standard deviations (numbers above the error bars) between three repeated measurements.

Figure 5 shows the signals obtained from the CIGS solar cell according to the changes in dose rates. The signal differences due to changes in dose rates are within 2%. The error bars (standard deviations between three repeated measurements) increase as the dose rate decreases. This indicates that our system has a high uncertainty for dose rates of 100 and 200 MU/min compared to the other dose rates. Especially, although at the lowest dose rate (100 MU/min), the error bar is relatively high compared with that at a dose rate of 600 MU/min; it is within 2%.

### 3.3. Energy Independence

**Figure 6:**
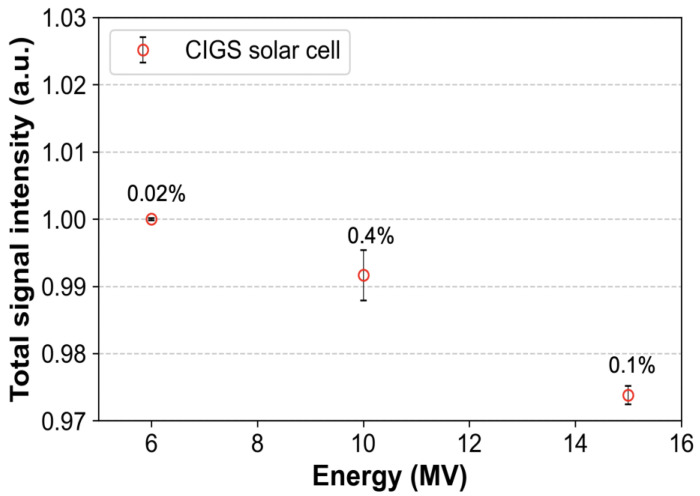
The signals obtained from the copper indium gallium selenide (CIGS) solar cell according to energy changes of the photon beam (6, 10, and 15 MV). Total signal intensity is the sum of the obtained signals from the CIGS solar cell during irradiation. All the signals were normalized based on the signal corresponding to the photon beam energy of 6 MV. Error bars denote the standard deviations (numbers above the error bars) between three repeated measurements.

Figure 6 shows that the signals obtained from the CIGS solar cell decrease as the energy of the photon beam increases. The difference in signal intensity between the 6 and 15 MV photon beam energies is within 3%, which indicates that the CIGS solar cell has an energy dependence on the photon beam. The error bars (standard deviations between three repeated measurements) are within 1% for each of the photon beam energies.

### 3.4. Field Size Output

**Figure 7:**
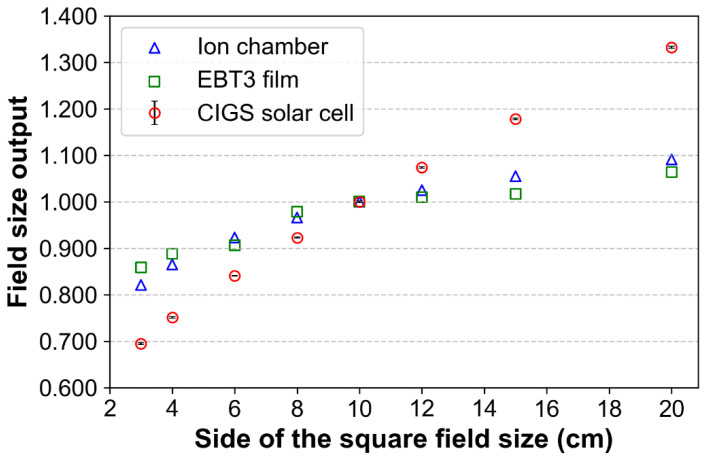
Field size output factors of copper indium gallium selenide (CIGS) solar cell, ion chamber, and film for various field sizes (3 × 3, 4 × 4, 6 × 6, 8 × 8, 10 × 10, 12 × 12, 15 × 15, and 20 × 20 cm^2^) in 6 MV photon beams. The normalization was performed based on the output factor corresponding to a field size of 10 × 10 cm^2^. Error bars denote the standard deviations between three repeated measurements.

Figure 7 shows the signals obtained from the CIGS solar cell, ion chamber, and film according to the changes in field sizes. The field size output of the CIGS solar cell increases as the field size increases. However, changes of output factor according to field sizes were relatively higher compared to that of ion chamber and film. This result indicates that the CIGS solar cell has a substantial field size dependence compared to the ion chamber and film. The error bars (standard deviations between three repeated measurements) approach zero for all field sizes.

### 3.5. Flexibility Test

**Figure 8:**
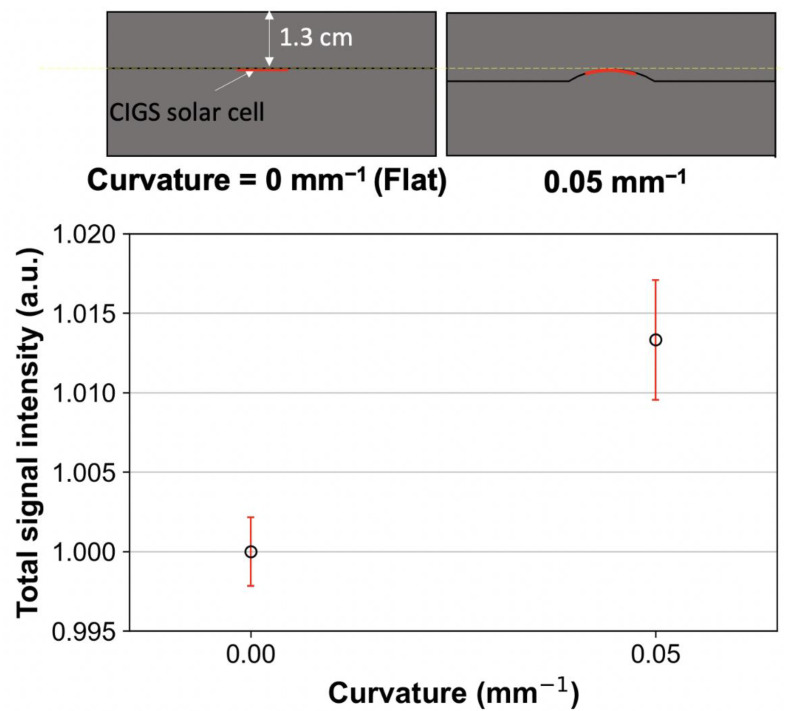
The copper indium gallium selenide (CIGS) solar cell signals under flat and bent conditions, respectively, in 6 MV photon beams. Zero curvature means a flat condition. The normalization was performed based on the signal corresponding to the flat CIGS solar cell. Error bars denote the standard deviations between three repeated measurements.

Figure 8 shows the signals of the CIGS solar cell in bent and flat conditions, respectively. The signal in the bent condition (0.05 mm^−1^ of curvature) was approximately 1.3% higher than that in the flat condition due to the set-up error of the CIGS solar cell. Additionally, the error bar in the bent condition was slightly higher than that on the flat condition (standard deviation: 0.4% for bent vs. 0.2% for flat). The error bars were within 1% for both conditions.

## 4. Discussion

This study is the first to report the dosimetric characteristics, such as dose linearity, dose rate independence, energy independence, and field size output, of a CIGS solar cell-based measurement system for therapeutic X-ray radiation using MV photon beams. Although our system exhibits higher dependence for field size output compared to that of the ion chamber, good dose linearity (the linearly fitted curve has a slope of 1.00 and R-square value of 0.9999) and dose rate independence (within 2%) were observed. These results demonstrate that our system has good characteristics as a flexible dosimeter for in vivo dosimetry.

Our system showed energy dependence (within 3%). That is, the signals of the CIGS solar cell decreased as energy increased. One main reason for this dependence may be due to the increase of backscatter from the stainless-steel sheet used for substrate (Figure 1). A previous study showed the backscatter depended on Compton scattering (primary photon) in high Z material [61]. Additionally, it is known as that Compton scattering at low MV photon beams is higher than that at high MV photon beams [62]. Potentially, the energy dependence can be improved by multiplying the weighting factors.

The field size output measured by our system is not similar to that by ion chamber and film, which could be attributed to energy dependence. In linear accelerators, photon beams with large field sizes typically have a higher number of low-energy photons than those with small field sizes [63]. Therefore, large field sizes can excessively increase the signal intensities of our system by a relatively large number of low-energy photons, despite an energy dependence of <3%. Unfortunately, this problem makes accurate dose measurement difficult in volumetric modulated arc therapy with variable field sizes. However, there is no major problem in the special radiotherapy techniques with fixed field sizes such as TBI. That is, in these techniques, the problem can potentially be overcome by correction using the fitted curve corresponding to the field size output (Figure 7).

**Figure 9:**
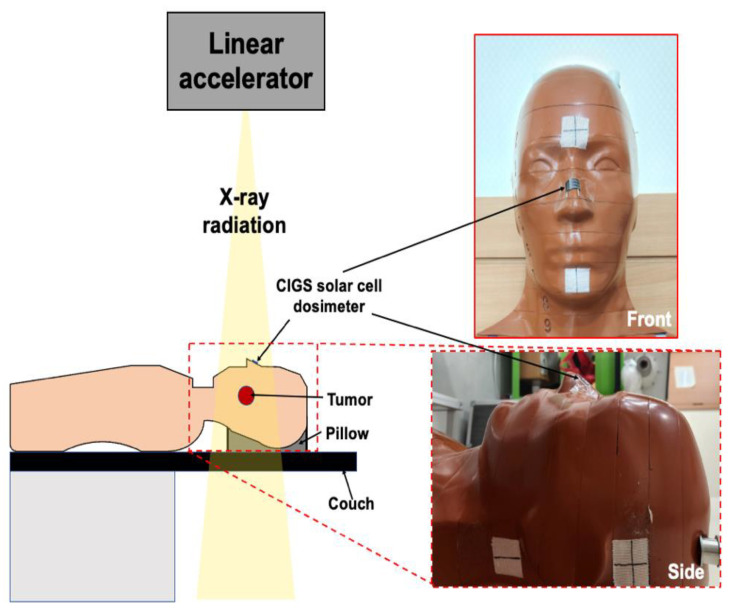
A simple example of in vivo dosimetry using our system based on copper indium gallium selenide (CIGS) solar cell. The CIGS solar cell was attached to the surface of the anthropomorphic phantom in the bent condition in this example.

Although the CIGS solar cell cannot simulate the perfect flexibility compared to flexible sensors based on hydrogel [64,65], there is no major problem in attaching it to the human surface, such as the back of the hand, thorax, and forehead. Furthermore, the signal of the CIGS solar cell in the bent condition (curvature radius of 20 mm) was comparable to that in the flat condition. Our measurement system has good characteristics such as real-time quantifying the absorbed dose compared to the flexible hydrogel sensors. The previously proposed hydrogel sensors can estimate the absorbed dose quickly by distinguishing colors with eyes [64,65]. However, an additional reading process is required for accurate quantification. On the contrary, our system can quantify the absorbed dose in real-time, which could be useful in clinics. Therefore, our system has the potential as a flexible real-time in vivo dosimeter. Figure 9 shows an example of in vivo dosimetry using our system.

The proposed dose measurement system based on the CIGS solar cell has two major differences from those of previously proposed flexible dosimeters based on a-Si solar cells [49,50]. The first is the easy customization of the size and shape of the solar cell. This feature enables the measurement area, i.e., the area on which high-energy photons are incident, of our system to be clinically acceptable (10 × 10 cm^2^). In contrast, previously proposed flexible a-Si solar cell dosimeters, despite being capable of point dose measurement, have relatively large sizes, such as 1.5 × 5.0 cm^2^ [49] and 2.0 × 2.0 cm^2^ [50], which could reduce clinical usefulness. The second is dose rate independence (within 2%), which is useful for measuring radiation doses delivered at different dose rates without correction. However, the previous dosimetry system (a-Si solar cell with a scintillator screen) exhibited a substantial dependence on the dose rate (within 7%) [50].

However, the proposed system has several limitations. One major limitation is point dose measurement, i.e., one-dimensional detection. To overcome this limitation, our potential future work includes a 2D array system based on strip-shaped CIGS solar cells. The array system comprises two layers arranged along the x- and y-coordinates in a manner similar to that of the optical fiber array system from our previous study [20]. A second limitation is the use of electric wires/cables for transmitting electrical signals from solar cells to the DAQ module, which can cause discomfort to patients during in vivo dosimetry for radiation therapy. This limitation can be overcome by using a wireless DAQ module, such as a radio-frequency transmitter and receiver, in a manner similar to that in the study by Zygmanske et al [49]. However, wireless transmission can adversely affect system performance in terms of the sampling rate. Therefore, it should be adopted according to the purpose of clinical use.

## 5. Conclusions

We proposed a real-time dose measurement system based on a flexible CIGS solar cell for therapeutic X-ray radiation. Our findings demonstrate that the proposed system has good dosimetric characteristics, such as dose linearity/dose rate independence. In addition, the size and shape of the CIGS solar cell in our system are easily customizable in contrast to other a-Si solar cell-based dosimeters. In conclusion, the proposed system could be a useful tool such as an in vivo dosimeter for total body irradiation.

## Figures and Tables

**Table 1:** Experimental variables to evaluate the dosimetric characteristics of our measurement system based on a copper indium gallium selenide solar cell.

Evaluations	Experimental Variables				
	Energy (MV)	Field Size (cm^2^)	Dose Rate (MU/min)	Irradiated Dose (cGy)	Measurement Depth (cm)
Dose linearity	6	10 × 10	600	20, 50, 100, 150, 200, 300, 500, 800, and 1200	d_max_
Dose rate independence	6	10 × 10	100–600	100	d_max_
Energy independence	6, 10, and 15	10 × 10	600	100	d_max_
Field size output	6	From 3 × 3 to 20 × 20	600	100	10
Flexibility test	6	10 × 10	600	100	1.3 (in HIPS material)

Abbreviations: d_max_ = depth at maximum dose; MU = monitor unit, HIPS = high impact polystyrene.

**Table 2:** Standard deviations between repeated three measurements for linearity evaluation.

Delivered dose (cGy)	20	50	100	150	200	300	500	800	1200
Standard deviation (cGy)	0.01	0.07	0.08	0.30	0.33	0.80	1.07	0.10	0.70

## Data Availability

The original contributions presented in the study are included in the article, and further inquiries can be directed to the corresponding authors.
